# “DEMENTIA-FRIENDLY”: WHAT DOES IT MEAN TO UNDERGRADUATE STUDENTS?

**DOI:** 10.1093/geroni/igac059.1712

**Published:** 2022-12-20

**Authors:** Nasreen Sadeq

**Affiliations:** University of South Florida, Tampa, Florida, United States

## Abstract

Experiential learning assignments may be a potential method of educating undergraduate students about relevant issues in gerontology. Experiential learning involves applying knowledge learned in the classroom to real-world situations and requires students to engage in reflection. The current study explores undergraduate students’ perspectives on what it means to be “dementia-friendly” through an activity completed outside of the classroom. Students enrolled in an Alzheimer’s Disease Management course completed a module about quality of life, which included information about dementia-friendly communities. Then, students visited a location of their choice that is often frequented by older adults. Based on what they learned in class, they explored the location and documented observations about its dementia-friendly characteristics, or lack thereof. Following their visit, students wrote a reflection on their experience and suggested ways to improve dementia-friendliness. Consensual qualitative methods were used to explore themes in student responses. Among students (N=56), the majority visited grocery or big-box stores, followed by retail shops and restaurants. The three most common themes that emerged about essential dementia-friendly characteristics were 1) availability of helpful people, 2) easy to read signs, and 3) easy to navigate with few fall hazards. Themes from students’ suggestions for improving dementia-friendliness included 1) items with larger font sizes, 2) providing staff with dementia training, and 3) more amenities (e.g., store maps, personal shoppers). Student reflections suggested that the assignment also increased awareness and empathy about challenges faced by older adults who wish to age in place.

